# The negative impact of long working hours on mental health in young Korean workers

**DOI:** 10.1371/journal.pone.0236931

**Published:** 2020-08-04

**Authors:** Sungjin Park, Hyungdon Kook, Hongdeok Seok, Jae Hyoung Lee, Daeun Lim, Dong-Hyuk Cho, Suk-Kyu Oh

**Affiliations:** 1 Department of Occupational and Environmental Medicine, Cheonan Medical Center, Cheonan, Korea; 2 Division of Cardiology, Department of Internal Medicine, Korea University Medical Center, Seoul, Korea; 3 Department of Occupational and Environmental Medicine, Busan Adventist Hospital, Sahmyook Medical Center, Busan, Korea; 4 Medical School for International Health, Ben Gurion University, Beersheba, Israel; 5 Department of Biological Sciences, University of Seoul, Seoul, Korea; Universitat de Valencia, SPAIN

## Abstract

Long working hours are known to have a negative effect on health. However, there is no clear evidence for a direct link between mental health and long working hours in the young adult populations. Therefore, we aimed to determine whether long working hours are associated with mental health in young adult workers. Data were collected from a 2012 follow-up survey of the Youth Panel 2007. A total of 3,332 young adult employees (aged 20 to 35) were enrolled in the study. We analyzed stress, depression, and suicidal thoughts by multivariate logistic regression analysis based on working hours (41 to 50, 51 to 60 and over 60 hours, compared to 31 to 40 hours per week), which was adjusted for sex, age, marriage status, region, and educational level. From the 3,332 young adult employees, about 60% of the workers worked more than 40 hours and 17% of the workers worked more than 50 hours per week. In a Chi-square test, stress level, depression, and suicidal thoughts increased with increasing working hours (p-value <0.001, 0.007, and 0.018, respectively). The multivariate logistic regression model showed that, compared to the 31 to 40 hours per week group, the adjusted odds ratios of the 41 to 50, 51 to 60, and over 60 hours per week groups for stress were 1.46(1.23–1.74), 2.25(1.79–2.83) and 2.55(1.72–3.77), respectively. A similar trend was shown in depression [odds ratios: 2.08(1.23–3.53), 2.79(1.44–5.39) and 4.09(1.59–10.55), respectively] and suicidal ideation [odds ratios: 1.98(0.95–4.10), 3.48(1.48–8.19) and 5.30(1.61–17.42), respectively]. We concluded that long working hours were associated with stress, depression, and suicidal ideation in young employees, aged 20 to 35.

## Introduction

Long working hours are known to affect health negatively. One of the reasons is the unhealthy behaviors associated with working overtime, such as increased alcohol consumption and lack of exercise [1, 2]. In addition, employees working long hours may not have the time to seek proper medical treatment when they fall ill [3]. Furthermore, working long hours may induce hypertension, diabetes, and metabolic syndrome and is closely linked to ischemic heart disease, stroke, and increased mortality [2, 4–7].

In addition to the effects on physical health, adverse effects on mental health were observed in employees working long hours. An excessive workload increases workers’ fatigue and thereby negatively affects the subjective perceptions of health [8, 9]. In addition, anxiety and depression are more common in the group working long hours [10].

Negative impact on mental health is manifested in many ways, including stress, depression, and suicidal ideation. These are important issues not only for the individual but also for the society. Depression may occur in people who are frequently exposed to stress, leading to development of disease and poor quality of life, and may eventually induce serious suicidal thoughts. In turn, suicidal impulses have a significant impact on the quality of mental and physical health and can contribute greatly to the worldwide disease burden [11].

Many countries aim to limit the working hours of their employees, as it is known that longer working hours have a negative impact on health [12]. Modern society tends to emphasize the quality of life, since people hope for stable employment with enough time for rest and leisure rather than simply earning a lot of money [13]. On the other hand, the time for leisure and rest are limited for employees with long working hours, which ultimately puts stress on individuals and may have a negative impact on their mental health [14–16].

According to the data obtained by the Organization for Economic Co-operation and Development (OECD), the average annual working hours in OECD countries has decreased from 1,881 hours in 1990 to 1,734 hours in 2018. These data also showed that the average annual working hours in South Korea decreased from 2,677 hours in 1990 to 1,993 hours in 2018, but this number is still high compared to that reported for other OECD countries [17]. As of 2018, South Korea has the third highest number of annual working hours among OECD countries following Mexico and Costa Rica. Compared with the 1,363 hours reported for Germany, the OECD country with the lowest number of annual working hours, South Korea has 1.5 times longer working hours.

Although negative effects of long working hours on the health of workers are already known, not many studies have been conducted to establish the association between long working hours and mental health with specific parameters such as depression and suicidal ideation, especially in Korea. We therefore aimed to investigate the impact of an excessive workload on mental health using the Youth Panel and its follow-up surveys.

In geriatric groups, it is hard to attribute a health problem to an independent factor because there are a lot of pre-existing medical conditions [18, 19]. We specifically investigated youth, because we could associate mental health with working hours as an independent variable. In addition, in Korea, the young adult population is the group that works the longest [20]. Therefore, this group is the most suitable for finding out the link between long working hours and mental health.

We examined the differences in stress levels, depression, and suicidal thoughts in the young adult population with respect to mental health problems resulting from long working hours.

## Methods

### Study population

We did a cross-sectional analysis of the data from the Youth Panel (hereafter “YP”) 2007. YP was conducted by Korea Employment Information Service (KEIS, https://survey.keis.or.kr/eng/yp/yp01.jsp). This panel survey was aimed to collect basic data on school life, social and economic activities, and household background of young people to contribute to the development of employment policies and related research in decreasing youth unemployment. The YP was a longitudinal survey initially conducted in 2007 with annual follow-up surveys until 2012. It was the first individual panel survey conducted on the Korean population. The YP 2007 surveyed 10,206 young people aged between 15 and 29 by establishing a nation-wide sample of young residents. Of the initial survey participants, 91.2% completed the second survey (first follow-up) in 2008; 86.5%, the third survey (second follow-up) in 2009; 81.7%, the fourth survey (third follow-up) in 2010; 78.9%, the fifth survey (fourth follow-up) in 2011; and 76.8%, the sixth survey (fifth follow-up) in 2012. In the sixth YP survey, 7,843 participants remained, of which 7,057 actually completed the survey as some participants did not respond to the whole questionnaire.

These surveys were conducted via interview visits using Computer-Assisted Personal Interview (CAPI). In addition, some questionnaires were also answered online (Computer-Assisted Web Interview, CAWI) or by telephone (Computer-Assisted Telephone Interview, CATI) in cases where the respondents rejected the survey method or had difficulty coming in for a personal interview. Of the total respondents, 94.6% used CAPI, 3.6% used CAWI, and 1.8% used CATI. The interview period lasted from August 6, 2012 to November 30, 2012, but for some responders, information was gathered in early December.

### Study design

**Fig 1:**
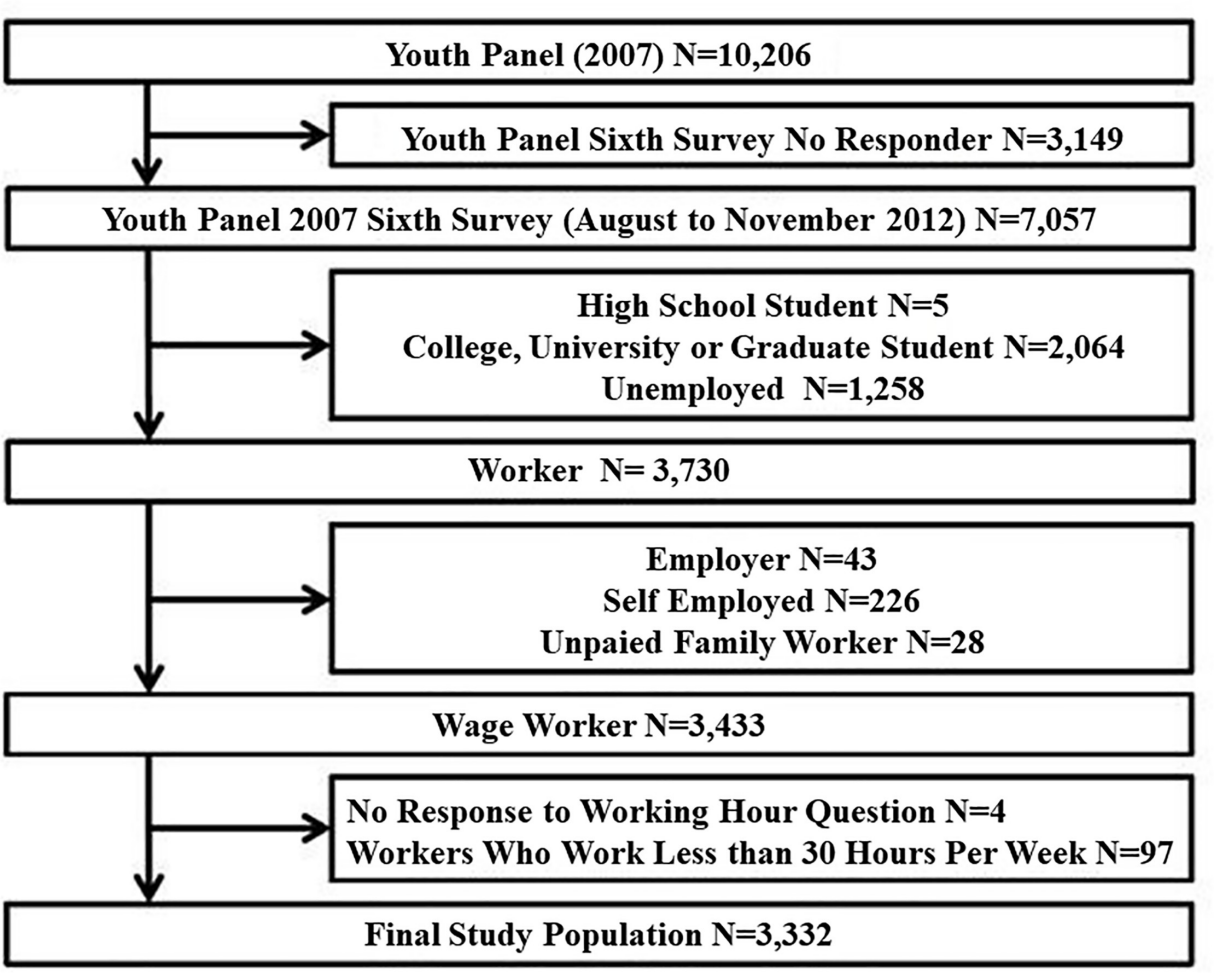
Study population & design.

This study was conducted on a sample of participants from the YP 2007. From the 10,206 individuals who took part in the initial survey, only 7,057 participants remained as 3,149 did not respond in 2012. Of the 7,057 remaining participants, 5 high school students, 2,064 college, university, or graduate students and 1,258 unemployed individuals were excluded. From this, a further 398 participants were excluded from the study: 43 employers, 226 self-employed, 28 unpaid family workers, 4 people who did not respond to the working hours question, and 97 participants who worked less than 30 hours per week and were not considered to be full-time employees in Korea [3, 4]. Finally, 3,332 participants were selected for the study (Fig 1). We excluded part-time workers because of their irregular working time, time-shifting and part-time working status itself on Korean social background could affect mental health [21]. Institutional Review Board of Korea University Medical Center Anam Hospital specially approved this study (IRB number: 2019-AN-0204).

### Working hours

Data on working hours were obtained from answers to economic activity questions listed in Part B of the sixth YP. This part of the survey contained questions on working hours, working days, and wages. For example, question B44 was as follows: "How many hours do you work on average per week in your current job?". Possible answers were "regular working hours per week: ___ hours" and "average overtime hours per week: ___ hours". The average working hours per week were calculated as the sum of regular working hours and average overtime hours per week. The average working hours per week were divided into increments of 10 hours, yielding four groups, namely “31 to 40 hours per week”, “41 to 50 hours per week”, “51 to 60 hours per week”, and “more than 60 hours per week” [3, 4].

### Mental health

Part H of the sixth YP was evaluated to characterize mental health issues. Question H22 was "How often do you feel stressed in your daily life?", with possible answers of “very often", “often", “sometimes", and " rarely". Answering "very often" and "often" was considered to indicate a high stress level, while participants answering "sometimes" and "rarely" were considered not feeling very stressed.

Depression was assessed in question H23: "Have you ever felt sadness or feeling of despair that interfered with your daily life for two consecutive weeks over the past year?", with possible answers being “yes” or “no.” Participants answering “yes” to this question were considered depressed, while those answering “no” were considered not depressed.

Suicidal ideation was identified in question H24: "Have you ever thought about wanting to die in the last year?", with participants answering “yes” classified as those with suicidal thoughts and ones answering “no” as those without suicidal thoughts.

### Covariates

Both men and women participants between the ages of 20 to 35 years were recruited. Marriage status was assessed as married, unmarried, or divorced. Residential areas were classified as special cities, metropolitan cities, or other provinces. The category assessing the highest level of education was divided into high school graduation or lower and college degree or higher.

### Statistical analysis

We conducted a chi-square test for general characteristics according to working hours, stress, depression, and suicidal ideation. We analyzed stress, depression, and suicidal ideation by multivariate logistic regression analysis generating an odds ratio (OR) and 95% confidence interval (CI) for those working between 41 hours and 50 hours, between 51 and 60 hours, and over 60 hours per week relative to those working between 31 and 40 hours with crude model and adjusted model with associated covariate such as sex, marriage, region, and educational level.

SAS software version 9.2 (SAS Inc., Cary, NC, USA) was used for statistical analysis and a p-value below 0.05 was considered to be statistically significant.

## Results

### General characteristics of participants relative to working hours

In the sixth YP, 1,339 (40.2%) workers responded to working between 31 and 40 hours per week, 1,425 (42.8%) between 41 and 50 hours, 450 (13.5%) between 51 and 60 hours, and 118 (3.5%) more than 60 hours (Table 1, S1 and S2 Tables).

**Table 1:** General characteristics of participants relative to working hours.

			Working hours, n (%)								
		Total	31–40		41–50		51–60		Over 60		p-value
Gender	Male	1629	569	(34.9)	702	(43.1)	275	(16.9)	83	(5.1)	<0.001
	Female	1703	770	(45.2)	723	(42.4)	175	(10.3)	35	(2.1)	
Marriage status	Married	707	290	(41.0)	296	(41.9)	99	(14.0)	22	(3.1)	0.812
	Unmarried or divorced	2625	1,049	(40.0)	1,129	(43.0)	351	(13.4)	96	(3.6)	
Residential area	Special or metropolitan city	2028	805	(39.7)	867	(42.7)	285	(14.1)	71	(3.5)	0.685
	Other province	1304	534	(40.9)	558	(42.8)	165	(12.7)	47	(3.6)	
Educational Level	High school graduation or below	701	232	(33.1)	273	(38.9)	145	(20.7)	51	(7.3)	<0.001
	College degree or above	2631	1,107	(42.1)	1,152	(43.8)	305	(11.6)	67	(2.5)	
Stress level	High	969	308	(31.8)	432	(44.6)	179	(18.5)	50	(5.1)	<0.001
	Low	2363	1,031	(43.6)	993	(42.0)	271	(11.5)	68	(2.9)	
Depression	Present	88	21	(23.9)	44	(50.0)	17	(19.3)	6	(6.8)	0.007
	Absent	3244	1,318	(40.6)	1,381	(42.6)	433	(13.3)	112	(3.5)	
Suicidal thoughts	Present	48	11	(22.9)	22	(45.9)	11	(22.9)	4	(8.3)	0.018
	Absent	3284	1,328	(40.4)	1,403	(42.7)	439	(13.4)	114	(3.5)	

In terms of stress levels, 23.0% of employees working between 31 and 40 hours per week responded that they felt a lot of stress, 30.3% of those working between 41 and 50 hours, 39.8% of those working between 51 to 60 hours, and 42.4% of those working more than 60 hours per week reported stress. Therefore, the proportion of workers who felt a lot of stress increased with increasing working hours.

Of those participants working between 31 and 40 hours per week, 1.6% had self-reported depression, while 3.1% of those working between 41 and 50 hours reported that they felt depressed, 3.8% of those working between 51 to 60 hours, and 5.1% of those working more than 60 hours per week reported depression. Hence, an increase in weekly working hours was associated with higher levels of depression.

A similar trend was observed for participants with suicidal thoughts over the past year (0.8% of those working between 31 and 40 hours, 1.5% of those working between 41 and 50 hours, 2.4% of those working between 51 to 60 hours, and 3.4% of those working more than 60 hours per week).

When analyzed by gender, 569 (42.5%) men and 770 (57.5%) women worked between 31 hours and 40 hours, 702 (49.3%) men and 723 (50.7%) women between 41 hours and 50 hours, 275 (61.1%) men and 175 (38.9%) women between 51 hours and 60 hours, and 83 (70.3%) men and 35 (29.7%) women more than 60 hours per week. Hence, the long working hours led to a higher proportion of men working compared to women.

In terms of the educational level, of the participants working between 31 and 40 hours, 232 (17.3%) were high school graduates or had a lower level of education and 1,107 (82.7%) had a college degree or a higher level of education, While this proportion was 273 (19.2%) to 1,152 (80.8%) in the group working between 41 hours and 50 hours. In the group of those working between 51 hours and 60 hours, 145 (32.2%) had a lower education level and 305 (67.8%) had a higher education level. While this proportion was 51 (43.2%) to 67 (56.8%) amongst those working more than 60 hours per week. Therefore, longer working hours led to a higher proportion of those with a lower education level.

### General characteristics of participants in terms of mental health

In the sixth follow-up survey of the YP 2007, 969 (29.1%) participants responded that they felt very stressed and 2,363 (70.9%) said they did not feel stressed. When analyzed by gender, 29.3% of males and 28.9% of females felt high levels of stress. (Table 2, S3 and S4 Tables)

**Table 2:** General characteristics of participants in terms of mental health.

		Stress level, n (%)					Depression, n (%)					Suicidal ideation, n (%)				
		High			Low	p-value	Present			Absent	p-value	Present			Absent	p-value
**Gender**	Male	477	(49.2)	1,152	(48.8)	0.833	31	(35.2)	1,598	(49.3)	0.013	14	(29.2)	1,615	(49.2)	0.009
	Female	492	(50.8)	1,211	(51.2)		57	(64.8)	1,646	(50.7)		34	(70.8)	1,669	(50.8)	
**Marriage status**	Married	204	(21.1)	503	(21.3)	0.918	15	(17.0)	692	(21.3)	0.402	5	(10.4)	702	(21.4)	0.096
	Unmarried or divorced	765	(78.9)	1,860	(78.7)		73	(83.0)	2,552	(78.7)		43	(89.6)	2,582	(78.6)	
**Residential area**	Special or metropolitan city	613	(63.3)	1,415	(59.9)	0.076	58	(65.9)	1,970	(60.7)	0.383	32	(66.7)	1,996	(60.8)	0.500
	Other province	356	(36.7)	948	(40.1)		30	(34.1)	1,274	(39.3)		16	(33.3)	1,288	(39.2)	
**Educational level**	High school graduation or below	208	(21.5)	493	(20.9)	0.734	19	(21.6)	682	(21.0)	1.000	12	(25.0)	689	(21.0)	0.617
	College degree or above	761	(78.5)	1,870	(79.1)		69	(78.4)	2,562	(79.0)		36	(75.0)	2,595	(79.0)	
**Working hours**	31 to 40	308	(31.8)	1,031	(43.6)	<0.001	21	(23.9)	1,318	(40.6)	0.007	11	(22.9)	1,328	(40.4)	0.018
	41 to 50	432	(44.6)	993	(42.0)		44	(50.0)	1,381	(42.6)		22	(45.9)	1,403	(42.7)	
	51 to 60	179	(18.5)	271	(11.5)		17	(19.3)	433	(13.3)		11	(22.9)	439	(13.4)	
	Over 60	50	(5.1)	68	(2.9)		6	(6.8)	112	(3.5)		4	(8.3)	114	(3.5)	

Eighty-eight (2.6%) of the interviewed employees reported that they felt depressed and 3,244 (97.4%) did not. Of all participants, 1.9% of men and 3.3% of women were depressed (p = 0.013).

Forty-eight (1.4%) participants answered that they had suicidal thoughts, and 3,284 (98.6%) responded that they did not. Of all participants, 0.9% of males and 2.0% of females had suicidal ideation, showing a statistically significant difference (p = 0.009).

There is no significant difference in stress, depression and suicidal ideation in terms of educational level, residential area, and marriage status.

Putting stress levels in relation to depression, only 27.7% of respondents who were not depressed felt very stressed. In contrast, 80.7% of depressed participants also felt very stressed (p<0.001). Most participants without suicidal ideation did not feel very stressed (28.3%), while the majority of participants with suicidal thoughts (83.3%) had high self-reported stress levels (p<0.001)

Putting self-reported depression in relation to stress levels, only 0.7% of the participants who did not feel stressed felt depressed, but of those reporting high stress levels, 7.3% also felt depressed (p<0.001). Only 1.8% of participants without suicidal thoughts were depressed. In contrast, 58.3% of participants with suicidal ideation had depression (p<0.001).

In terms of stress levels, only 0.3% of those not feeling stressed had suicidal thoughts, but 4.1% of those with high stress levels stated that they thought of suicide (p<0.001). Only 0.6% of the employees without depression responded that they had suicidal thoughts. In contrast, 31.8% of participants with depression had suicidal ideation (p<0.001).

### Relationship between working hours and mental health

**Fig 2:**
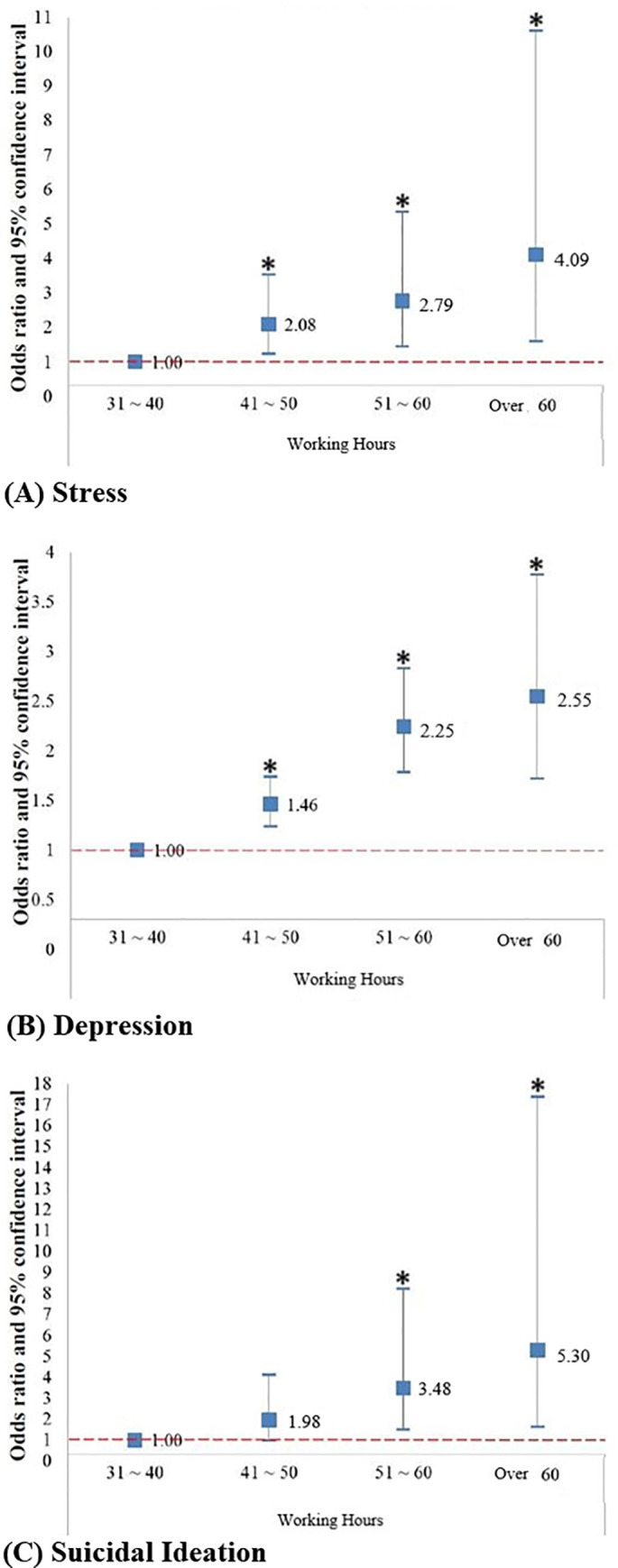
Relationship between working hours and mental health.

In the crude model, we determined the OR (95% CI) of experiencing high stress levels in terms of working hours. The OR was 1.46 (95% CI: 1.23–1.73) for employees working between 41 and 50 hours per week, 2.21 (95% CI: 1.76–2.78) for those working between 51 and 60 hours, and 2.46 (95% CI: 1.67–3.62) for those working over 60 hours, relative to those working between 31 and 40 hours per week (Table 3 and Fig 2).

**Table 3:** The association between working hours and mental health.

		Stress				Depression				Suicidal ideation			
		Odd Ratio (95% Confidence Interval)				Odd Ratio (95% Confidence Interval)				Odd Ratio (95% Confidence Interval)			
		Crude		Adjusted model		Crude		Adjusted model		Crude		Adjusted Model	
**Working Hours**	31–40	1.00		1.00		1.00		1.00		1.00		1.00	
	41–50	1.46	(1.23–1.73)	1.46	(1.23–1.74)	2.00	(1.18–3.38)	2.08	(1.23–3.53)	1.89	(0.91–3.92)	1.98	(0.95–4.10)
	51–60	2.21	(1.76–2.78)	2.25	(1.79–2.83)	2.46	(1.29–4.71)	2.79	(1.44–5.39)	3.03	(1.30–7.03)	3.48	(1.48–8.19)
	>60	2.46	(1.67–3.62)	2.55	(1.72–3.77)	3.36	(1.33–8.50)	4.09	(1.59–10.55)	4.24	(1.33–13.52)	5.30	(1.61–17.42)

* Adjusted model 1: 1 adjusted for gender, marriage status, residential area, and educational level.

The OR (95% CIs) for the depression in terms of working hours were 2.00 (95% CI: 1.18–3.38) for employees working between 41 and 50 hours per week, 2.46 (95% CI: 1.29–4.71) for those working between 51 and 60 hours, and 3.36 (95% CI: 1.33–8.50) for those working more than 60 hours per week, relative to employees working between 31 and 40 hours per week.

In suicidal ideation, the OR 1.89 (95% CI: 0.91–3.92) was obtained in crude model for the employees working between 41 and 50 hours per week, 3.03 (95% CI: 1.30–7.03) for those working between 51 and 60 hours per week, and 4.24 (95% CI: 1.33–13.52) for those working more than 60 hours per week, relative to employees working between 31 and 40 hours.

In adjusted model, which was adjusted for gender, marriage status, region, and educational level, we determined the following ORs (95% CI) of experiencing high-stress level: 1.46 (95% CI: 1.23–1.74) for employees working between 41 and 50 hours per week, 2.24 (95% CI: 1.78–2.83) for those working between 51 and 60 hours, and 2.55 (95% CI: 1.72–3.77) for those working over 60 hours, relative to those working between 31 and 40 hours.

Using adjusted model, the OR (95% CI) for the depression was 2.09 (95% CI: 1.23–3.54) for those working between 41 to 50 hours per week, 2.77 (95% CI: 1.43–5.35) for those working between 51 and 60 hours, and 4.11 (95% CI: 1.54–10.60) for those working over 60 hours a week, relative to employees working between 31 and 40 hours per week.

In analysis of suicidal ideation with adjusted model, the OR for employees working between 41 and 50 hours per week was 1.97 (95% CI: 0.95–4.09), 3.49 (95% CI: 1.48–8.23) for those working between 51 and 60 hours, and 5.28 (95% CI: 1.61–17.36) for those working over 60 hours per week, relative to those working between 31 and 40 hours per week.

## Discussion

In this study, we investigated the association between working hours and mental health. We revealed that as the working hours increase, the risk of stress, depression and suicide ideation tends to increase. All of these three mental health parameters had a linear dose response pattern correlating with increasing working hours.

In assessing the general characteristics of study participants in terms of their respective working hours, we discovered that the proportion of male employees increased with increasing working hours. This may reflect the fact that women typically take on multiple roles including housework and cannot commit as many hours to their employment as their male counterparts. Similar results were obtained in previous studies [22].

The increase in working hours was proportional to the stress levels in a dose-response manner, in that more work led to higher observed stress levels. These results remained the same after adjusting for sex, age, marriage status, region, and educational level. We observed that an increase in working hours, particularly those that were unintentional or unwanted, leads to higher stress levels in the employees. Stress in turn leads to unhealthy behaviors [23–25] and is known as a link to many diseases [26]. High stress levels induced by long working hours are known to influence unhealthy behaviors such as lack of exercise, higher smoking rate, and increased alcohol consumption [23–25]. In addition, stress not only affects the subjective health status and sleep duration but also impairs mental health, leading to depression or even suicidal thoughts [27, 28]. Stress due to long working hours is one of the major causes of health deterioration.

Significant differences were observed in the depression between sexes, in agreement with previous studies [29]. As mentioned above, depression has been shown to be related to subjective health status, sleep duration, and stress [27, 28]. A dose-response relationship was observed between depression and working hours in that employees with longer working hours increasingly reported feeling depressed. These results did not change after adjusting for sex, age, marriage status, region, and educational level. In agreement with previous studies, we determined that extended working hours, particularly those that are unintentional or unwanted, result in more depression [14, 15].

Women tended to have suicidal ideation more frequently than men, which corroborates the results of previous studies [30]. Suicidal ideation is known to be linked to subjective health status, sleep duration, stress, and depression [31]. Suicidal ideation was related to working hours in a dose-response manner, as the rate of depression increased with increasing working hours. These results were maintained after adjustment for gender, age, marriage status, region, and educational level. We conclude that extended working hours cause stress, which in turn may lead to depression or even suicide. These results are consistent with previous studies [14, 16].

Previous studies showed the association of depression and anxiety with working hours. Shields et al. showed that depression was increased in women who worked over 40 hours per week [32]. Workers were divided into only two groups based on 40 hours per week work hours. Therefore, that study could not show a dose-response pattern of working hours and increasing mental health problems. Virtanen et al. showed that depression and anxiety were increased in employees working over 55 hours per week compared with the 35 to 40 hours group [10]. However, working hours were divided into 3 groups and employees who worked 41 to 55 hours per week did not show significant difference in both depression and anxiety. Kim et al. also showed a similar result that employees who worked over 60 hours per week had more depressive symptoms than those working less than 52 hours [14]. However, there was no significant difference in the 52 to 60 hours per week group. Our study divided the working hours into more groups, which allows for a more significant conclusion that reducing working hours leads to reduced depression.

There were also investigations done on the relationship between suicidal ideation and working hours. Kim et al. showed that suicidal ideation was more frequent in over 60 working hours per week group than in the 40 to 51 working hours group [33]. Interestingly, this study pointed out that less than 40 working hours per week group had numerically more frequent suicidal thoughts compared to the 40 to 51 working hours group. Yoon et al also demonstrated that the risk of suicidal ideation increased in over 60 working hours per week group compared with less than 52 working hours group [16]. However, this study also failed to prove that more working hours leads to more frequent suicidal ideation. Compared with the other studies, our study showed a dose-response relationship between working hours and suicidal ideation. In our study, it was confirmed that about 60% of the employees work more than 40 hours a week, and 17% work more than 50 hours a week in the Korean young adult population. As the proportion of working overtime was high, working time could be stratified into four groups confirming the quantitative relationship between working hours and mental health. In previous studies of Korean employees, working hours were divided in a similar way and the result was analyzed by stratified groups [3, 4].

Many hypotheses have been put forward to explain the negative effects of extended working hours on physical and mental health [15]. Long hours at work necessitate sufficient recovery from fatigue, but the amount of time spent at home is limited [34]. This in turn leads to insufficient sleep. Even after adjusting the leisure time of individual workers, long working hours deteriorate the quality and duration of sleep [35]. Insufficient sleep is known to be associated with mental illnesses such as depression and anxiety and makes recovery impossible with detrimental effects on physical health [27, 36, 37].

In terms of suicidal ideation, same detrimental effect could be applied. Long working hours not only increase physical, psychological, and emotional demand of recovery, but also decrease the recovery time. [2, 34] Working hours also affect the quality and duration of sleep [35]. Therefore, long working hours aggravate anxiety, depression and burnout and, as a result, long working hours increase suicidal ideation [16].

Another hypothesis to explain the negative effects of long working hours on health incorporates the imbalance models of demand and adjustment and of effort and compensation [38, 39]. Long hours at work reflect an existing demand for work, but an individual worker cannot adjust such working environments or situations. If there is no adequate compensation for overtime work, stress and depression may ensue [15].

The strength of our study lies in the fact that it is a large-scale epidemiological study with over 3,300 wage workers based on panel surveys. In addition, participants of the YP are aged between 20 and 35 and are comparatively similar to each other, thereby reducing errors caused by age differences. Furthermore, we based our evaluation on the YP and therefore had the advantage of identifying the influence of independent variables on health in a particular age group. With increasing age, it becomes more difficult to attribute health problems to precise independent variables because of increasing incidences of underlying chronic diseases. In our study, all participants were 35 years old or younger and we could investigate mental health with working hours as the independent variable. In addition, we were able to evaluate all three typical indicators of mental health, namely stress, depression, and suicidal thoughts, which is more meaningful than analyzing only one indicator. Another strength of our study is the division of workload per week into four groups (31 hours to 40 hours, 41 hours to 50 hours, 51 hours to 60 hours, and over 60 hours work per week), rendering results that identified dose-response relationships.

Nevertheless, this study has some limitations. This study was based on the YP 2007 but was designed as a cross-sectional study assessing only the sixth follow-up survey of the panel. This cross-sectional approach has the disadvantage of making a precise causal relationship difficult to confirm. Furthermore, the independent variables of this study were obtained with a questionnaire and were therefore self-reported, which may have caused recall bias or reporting bias. Nevertheless, it is challenging to accurately calculate the actual working hours, and most similar studies have used individual questionnaires as well [3, 14, 16]. The three indicators of mental health were also self-reported reflecting the subjective information on the individual’s health status and did not include sufficient objective information. Nevertheless, we can overcome these limitations by focusing on variables such as subjective health and mental health. Another limitation is that we performed statistical analysis with adjustment to gender rather than stratification of gender, even though there were significant differences of mental problems in each gender. Finally, in our study, the prevalence of depression and suicidal ideation was 2.6% and 1.4%, respectively. This low percentage could render certain outcomes unstable.

## Conclusion

Our study has shown that an extensive weekly workload has a negative impact on mental health. We discovered that the longer working hours, the higher level of stress and higher prevalence of depression and suicidal ideation. All the three mental health parameter showed dose response pattern with working hours. We concluded that long working hours were associated with stress, depression, and suicidal ideation in young employees, aged 20 to 35.

## Supporting information

S1 File (ZIP)

S1 Table General characteristics of participants relative to working hours in males. (DOCX)

S2 Table General characteristics of participants relative to working hours in females. (DOCX)

S3 Table General characteristics of participants relative to mental health in males. (DOCX)

S4 Table General characteristics of participants relative to mental health in female. (DOCX)
