# Supplementary material for: The negative impact of long working hours on mental health in young Korean workers
Source: PLoS One. 2020 Aug 4;15(8):e0236931. doi: 10.1371/journal.pone.0236931 (PMC7402483; doi:10.1371/journal.pone.0236931)
Supplement: S1 Table — (DOCX) [file pone.0236931.s002.docx]

S1 Table General characteristics of participants relative to working hours in males

|  |  | Working hours, n (%) | | | | | | | |  |
| --- | --- | --- | --- | --- | --- | --- | --- | --- | --- | --- |
|  |  | 31-40 | | 41-50 | | 51-60 | | Over 60 | | p-value |
| Marriage status | Married | 143 | (33.9) | 187 | (44.3) | 76 | (18.0) | 16 | (3.8) | 0.440 |
|  | Unmarried or divorced | 426 | (35.3) | 515 | (42.7) | 199 | (16.5) | 67 | (5.5) |  |
| Residential area | Special or metropolitan city | 333 | (34.2) | 408 | (41.9) | 183 | (18.8) | 50 | (5.1) | 0.093 |
|  | Other province | 236 | (36.0) | 294 | (44.9) | 92 | (14.1) | 33 | (5.0) |  |
| Educational Level | High school graduation or below | 114 | (27.6) | 160 | (38.7) | 99 | (24.0) | 40 | (9.7) | <0.001 |
|  | College degree or above | 455 | (37.4) | 542 | (44.6) | 176 | (14.5) | 43 | (3.5) |  |
| Stress level | High | 131 | (27.5) | 202 | (42.3) | 118 | (24.7) | 26 | (5.5) | <0.001 |
|  | Low | 438 | (38.0) | 500 | (43.4) | 157 | (13.6) | 57 | (5.0) |  |
| Depression | Present | 5 | (16.1) | 10 | (32.3) | 11 | (35.5) | 5 | (16.1) | <0.001 |
|  | Absent | 564 | (35.3) | 692 | (43.3) | 264 | (16.5) | 78 | (4.9) |  |
| Suicidal thoughts | Present | 5 | (35.7) | 3 | (21.4) | 4 | (28.6) | 2 | (14.3) | 0.170 |
|  | Absent | 564 | (34.9) | 699 | (43.3) | 271 | (16.8) | 81 | (5.0) |  |
