# Supplementary material for: The negative impact of long working hours on mental health in young Korean workers
Source: PLoS One. 2020 Aug 4;15(8):e0236931. doi: 10.1371/journal.pone.0236931 (PMC7402483; doi:10.1371/journal.pone.0236931)
Supplement: S2 Table — (DOCX) [file pone.0236931.s003.docx]

S2 Table General characteristics of participants relative to working hours in females

|  |  | Working hours, n (%) | | | | | | | |  |
| --- | --- | --- | --- | --- | --- | --- | --- | --- | --- | --- |
|  |  | 31-40 | | 41-50 | | 51-60 | | Over 60 | | p-value |
| Marriage status | Married | 147 | (51.6) | 109 | (38.2) | 23 | (8.1) | 6 | (2.1) | 0.106 |
|  | Unmarried or divorced | 623 | (43.9) | 614 | (43.3) | 152 | (10.7) | 29 | (2.1) |  |
| Residential area | Special or metropolitan city | 472 | (44.8) | 459 | (43.5) | 102 | (9.7) | 21 | (2.0) | 0.591 |
|  | Other province | 298 | (45.9) | 264 | (40.7) | 73 | (11.2) | 14 | (2.2) |  |
| Educational Level | High school graduation or below | 118 | (40.9) | 113 | (39.3) | 46 | (16.0) | 11 | (3.8) | <0.001 |
|  | College degree or above | 652 | (46.1) | 610 | (43.1) | 129 | (9.1) | 24 | (1.7) |  |
| Stress level | High | 177 | (36.0) | 230 | (46.7) | 61 | (12.4) | 24 | (4.9) | <0.001 |
|  | Low | 593 | (49.0) | 493 | (40.7) | 114 | (9.4) | 11 | (0.9) |  |
| Depression | Present | 16 | (28.1) | 34 | (59.6) | 6 | (10.5) | 1 | (1.8) | 0.047 |
|  | Absent | 754 | (45.8) | 689 | (41.8) | 169 | (10.3) | 34 | (2.1) |  |
| Suicidal thoughts | Present | 6 | (17.6) | 19 | (55.9) | 7 | (20.6) | 2 | (5.9) | 0.004 |
|  | Absent | 764 | (45.8) | 704 | (42.2) | 168 | (10.0) | 33 | (2.0) |  |
