# Supplementary material for: The negative impact of long working hours on mental health in young Korean workers
Source: PLoS One. 2020 Aug 4;15(8):e0236931. doi: 10.1371/journal.pone.0236931 (PMC7402483; doi:10.1371/journal.pone.0236931)
Supplement: S3 Table — (DOCX) [file pone.0236931.s004.docx]

|  |  | Stress level, n (%) | | | | | Depression, n (%) | | | | | Suicidal thoughts, n (%) | | | | | |
| --- | --- | --- | --- | --- | --- | --- | --- | --- | --- | --- | --- | --- | --- | --- | --- | --- | --- |
|  |  | High | | Low | | p-value | Present | | Absent | | p-value | Present | | Absent | | p-value |  |
| Marriage status | Married | 136 | (28.5) | 286 | (24.8) | 0.138 | 7 | (22.6) | 415 | (26.0) | 0.826 | 2 | (14.3) | 420 | (26.0) | 0.490 |  |
|  | Unmarried or divorced | 341 | (71.5) | 866 | (75.2) |  | 24 | (77.4) | 1183 | (74.0) |  | 12 | (85.7) | 1195 | (74.0) |  |  |
| Residential area | Special or metropolitan city | 296 | (62.1) | 678 | (58.9) | 0.253 | 23 | (74.2) | 951 | (59.5) | 0.143 | 8 | (57.1) | 966 | (59.8) | 1.000 |  |
|  | Other province | 181 | (37.9) | 474 | (41.1) |  | 8 | (25.8) | 647 | (40.5) |  | 6 | (42.9) | 649 | (40.2) |  |  |
| Educational level | High school graduation or below | 119 | (24.9) | 294 | (25.5) | 0.858 | 8 | (25.8) | 405 | (25.3) | 1.000 | 4 | (28.6) | 409 | (25.3) | 1.000 |  |
|  | College degree or above | 358 | (75.1) | 858 | (74.5) |  | 23 | (74.2) | 1193 | (74.7) |  | 10 | (71.4) | 1206 | (74.7) |  |  |
| Working hours | 31 to 40 | 131 | (27.5) | 438 | (38.0) | <0.001 | 5 | (16.1) | 564 | (35.3) | <0.001 | 5 | (35.7) | 564 | (34.9) | 0.170 |  |
|  | 41 to 50 | 202 | (42.3) | 500 | (43.4) |  | 10 | (32.3) | 692 | (43.3) |  | 3 | (21.4) | 699 | (43.3) |  |  |
|  | 51 to 60 | 118 | (24.7) | 157 | (13.6) |  | 11 | (35.5) | 264 | (16.5) |  | 4 | (28.6) | 271 | (16.8) |  |  |
|  | Over 60 | 26 | (5.5) | 57 | (5.0) |  | 5 | (16.1) | 78 | (4.9) |  | 2 | (14.3) | 81 | (5.0) |  |  |

S3 Table General characteristics of participants relative to mental health in males
