# Supplementary material for: The negative impact of long working hours on mental health in young Korean workers
Source: PLoS One. 2020 Aug 4;15(8):e0236931. doi: 10.1371/journal.pone.0236931 (PMC7402483; doi:10.1371/journal.pone.0236931)
Supplement: S4 Table — (DOCX) [file pone.0236931.s005.docx]

S4 Table General characteristics of participants relative to mental health in females

|  |  | Stress level, n (%) | | | | | Depression, n (%) | | | | | Suicidal thoughts, n (%) | | | | | |
| --- | --- | --- | --- | --- | --- | --- | --- | --- | --- | --- | --- | --- | --- | --- | --- | --- | --- |
|  |  | High | | Low | | p-value | Present | | Absent | | p-value | Present | | Absent | | p-value |  |
| Marriage status | Married | 68 | (13.8) | 217 | (17.9) | 0.048 | 8 | (14.0) | 277 | (16.8) | 0.708 | 3 | (8.8) | 282 | (16.9) | 0.310 |  |
|  | Unmarried or divorced | 424 | (86.2) | 994 | (82.1) |  | 49 | (86.0) | 1369 | (83.2) |  | 31 | (91.2) | 1387 | (83.1) |  |  |
| Residential area | Special or metropolitan city | 317 | (64.4) | 737 | (60.9) | 0.187 | 35 | (61.4) | 1019 | (61.9) | 1.000 | 24 | (70.6) | 1030 | (61.7) | 0.381 |  |
|  | Other province | 175 | (35.6) | 474 | (39.1) |  | 22 | (38.6) | 627 | (38.1) |  | 10 | (29.4) | 639 | (38.3) |  |  |
| Educational level | High school graduation or below | 89 | (18.1) | 199 | (16.4) | 0.450 | 11 | (19.3) | 277 | (16.8) | 0.757 | 8 | (23.5) | 280 | (16.8) | 0.419 |  |
|  | College degree or above | 403 | (81.9) | 1012 | (83.6) |  | 46 | (80.7) | 1369 | (83.2) |  | 26 | (76.5) | 1389 | (83.2) |  |  |
| Working hours | 31 to 40 | 177 | (36.0) | 593 | (49.0) | <0.001 | 16 | (28.1) | 754 | (45.8) | 0.047 | 6 | (17.6) | 764 | (45.8) | 0.004 |  |
|  | 41 to 50 | 230 | (46.7) | 493 | (40.7) |  | 34 | (59.6) | 689 | (41.8) |  | 19 | (55.9) | 704 | (42.2) |  |  |
|  | 51 to 60 | 61 | (12.4) | 114 | (9.4) |  | 6 | (10.5) | 169 | (10.3) |  | 7 | (20.6) | 168 | (10.0) |  |  |
|  | Over 60 | 24 | (4.9) | 11 | (0.9) |  | 1 | (1.8) | 34 | (2.1) |  | 2 | (5.9) | 33 | (2.0) |  |  |
